# Construction and Performance Characterization of Hexahydro-1,3,5-trinitro-1,3,5-triazine/Poly(3,4-ethylene-dioxythiophene)–Poly(styrenesulfonate) Energetic Composites

**DOI:** 10.3390/molecules30051000

**Published:** 2025-02-21

**Authors:** Zhiwei He, Gongzhen Zhang, Chuanhao Xu, Wenyu Zhu, Jiawei Yue, Shengtao Zhou, Zhenyi Huang

**Affiliations:** 1 School of Chemical and Blasting Engineering, Anhui University of Science and Technology, Huainan 232001, China; 2022201175@aust.edu.cn (J.Y.); 2022201166@aust.edu.cn (S.Z.); 2022201224@aust.edu.cn (Z.H.); 2 Henan Huatong Chemical Industry Co., Ltd., Xinyang 465200, China; 13865542329@163.com; 3 School of Environment and Safety Engineering, North University of China, Taiyuan 030051, China; 4 Anhui Jiangnan Chemical Industry Co., Ltd., Hefei 230094, China; 17775210572@163.com

**Keywords:** RDX explosive, PEDOT:PSS, conductive polymer, mechanical properties, electrostatic spark sensitivity

## Abstract

Hexahydro-1,3,5-trinitro-1,3,5-triazine (RDX), a typical representative of energetic materials, is widely applied in military and industrial fields with its high energy density and excellent detonation performances. However, when used as a raw material for propellants and rocket propellants, RDX poses certain safety concerns due to its high sensitivity to external stimuli such as electrostatic discharge, impact, and friction, which limits its further application. Herein, to reduce the RDX electrostatic spark and mechanical sensitivities and improve safety performances, a conductive polymer of poly(3,4-ethylene-dioxythiophene)–poly(styrenesulfonate) (PEDOT:PSS) was introduced into the energetic material system based on a simple suction filtration method. RDX-based energetic composites with varying PEDOT:PSS mass fractions were prepared by both micron-sized RDX and nanosized RDX. The RDX-based energetic composites were characterized, and their response characteristics and performances were tested and compared. The results demonstrated that the conductive interfaces constructed by PEDOT:PSS on the RDX surface significantly reduced the electrostatic spark and mechanical sensitivity. The electrostatic spark sensitivity of μ-RDX-based energetic composites decreased by 40%, while the impact sensitivity and friction sensitivity decreased by 76.47% and 50%, respectively. Compared to micron-sized RDX-based energetic composites, the nano-sized RDX-based energetic composites desensitization effect on electrostatic spark sensitivity was more pronounced. For n-RDX-based energetic composites, the electrostatic spark sensitivity decreased by 66.4%. Furthermore, the assembly and desensitization mechanism of the RDX-based energetic composites were thoroughly investigated. This study not only provides a simple and reliable assembly method for the safe application of RDX but also offers corresponding data and experimental support for future research, which is of significant importance for the application of energetic materials.

## 1. Introduction

Energetic materials are metastable substances characterized by their high-power energy-release properties. Upon external stimulation, they can rapidly decompose, releasing substantial amounts of heat and gaseous products. They serve as energy sources for aerospace propulsion and weapon systems to execute predetermined actions, and they also form an important material basis for mineral development [1,2,3]. High explosives are typical energetic materials that are generally composed of low-mass organic molecules. This organic material primarily consists of energetic molecules as basic energy storage units that form macroscopically visible energetic crystals through ordered arrangements between the molecules. When these energetic crystals undergo micro/nanofabrication, they are referred to as micro–nano energetic crystals. However, it is important to note that while micro–nano energetic crystals enhance the reactive activity and improve mechanical safety, their electrostatic safety can be significantly reduced; herein, the reactive activity refers to the ease of decomposition and the intensity of the thermal reaction rate of energetic materials during thermal performance testing. This is due to the small physical size and low conductivity of micro–nano energetic crystals [4], which leads to local charge accumulation within them. When this charge accumulates above a threshold, an electrostatic discharge can occur, leading to chemical reactions in the energetic crystals and potentially causing accidents. Therefore, reducing the sensitivity to electrostatic sparks has become a significant issue in the field of micro–nano energetic crystal research, and constructing suitable conductive interfaces is a key approach to addressing this issue [5,6,7,8,9].

Based on this idea, researchers have attempted to incorporate inorganic carbon materials with good electrical conductivities, such as fullerenes, carbon black, and carbon nanotubes, into energetic materials. By applying the concept of material composites, electronic transport pathways have been constructed at the interface of energetic materials that effectively facilitate charge dissipation and significantly reduce the electrostatic spark sensitivity [10,11]. However, it is important to note that carbon materials generally lack reactive functional groups, which makes it difficult to form strong interactions with energetic molecules. While the adhesive properties of polymers can facilitate the combination of energetic crystals and carbon materials, this complicates the system and raises compatibility issues associated with the components. To address these issues, researchers have chosen graphene oxide (GO), which possesses reactive functional groups and a two-dimensional structure, as a functional material to construct conductive interfaces within micro–nano energetic crystals [2,12,13]. However, the carboxyl groups on the GO molecules impart a degree of electronegativity [2,3], which creates significant repulsive forces with the nitro groups on the energetic crystals. This necessitates the introduction of polymer materials or functional modification of the energetic crystals during the combination process to enhance the interfacial interactions. Therefore, to construct conductive interfaces on the surfaces of micro–nano energetic crystals, it is essential to first identify suitable conductive materials.

The molecular structure of conductive polymers contains conjugated carbon bonds formed by alternating single and double bonds, allowing them to maintain flexibility while exhibiting electrical conductivities similar to those of semiconductors and metals [14]. Leveraging this advantage, conductive polymers have garnered significant attention in fields such as coating materials [15], electrode materials [16], supercapacitors [17], sensors [18], and electromagnetic shielding [19], while providing new insights for constructing conductive interfaces in micro–nano energetic materials. For instance, Gibot et al. [20] introduced the conductive polymer polypyrrole (PPy) as an additive into an Al/SnO_2_ nano-aluminum thermite system, thereby decreasing the electrostatic spark sensitivity. This phenomenon also appears in a study examining Al/SnO_2_-conductive polymer polyaniline (PANI) [21], indicating that the introduction of conductive polymers can effectively reduce charge accumulation within micro–nano energetic materials, making them an important focus of research on sensitivity reduction for energetic materials, especially in the study of high explosives. However, regrettably, although researchers have studied the incorporation of PANI and similar materials into the energetic crystal octahydro-1,3,5,7-tetranitro-1,3,5,7-tetrazocine (HMX), successfully producing HMX-N@PANI and HMX/F2602-PANI composites with good electrostatic safety characteristics [22,23,24,25], overall, the development of this research direction has been relatively slow, with limited numbers of studies being reported.

In this study, the energetic crystal hexahydro-1,3,5-trinitro-1,3,5-triazine (RDX) and the conductive polymer poly(3,4-ethylene-dioxythiophene)–poly(styrenesulfonate) (PEDOT:PSS) were selected as research subjects. By utilizing the differential pressure generated by suction filtration, PEDOT:PSS was applied to the surface of RDX, resulting in the formation of RDX/PEDOT:PSS energetic composites with core–shell structures. Their morphologies, crystal structures, vacuum stabilities, thermal decomposition properties, and mechanical sensitivities were established. This research not only achieved the construction of conductive interfaces for micro–nano energetic crystals and expanded the direction of research into energetic crystal-conductive polymers but also provides an experimental basis that leverages the advantageous properties of nano-energetic crystals, with potentially significant practical application value.

## 2. Results and Discussion

### 2.1. Morphology, Microstructure, and Assembly Mechanism of RDX-Based Energetic Composites

Visually, the RDX appeared as a white powder (Figure S1). When loaded with varying amounts of PEDOT:PSS, the resulting energetic composite ranged in color from gray to dark gray, with a pronounced deepening of color that was positively correlated with the mass fraction of PEDOT:PSS. Because RDX is inherently a nonconductive material, it tends to accumulate static electricity through friction with other materials or its own particles, leading to significant electrostatic accumulation. This was particularly evident because the white RDX particles readily adhered to the inner walls of the sample vial. Interestingly, as shown in Figure S1, virtually no electrostatic adsorption of the RDX-based energetic composite was observed on the inner walls of the sample vial, indicating that the introduction of PEDOT:PSS effectively reduced the static charge accumulation of RDX.

**Figure 1:**
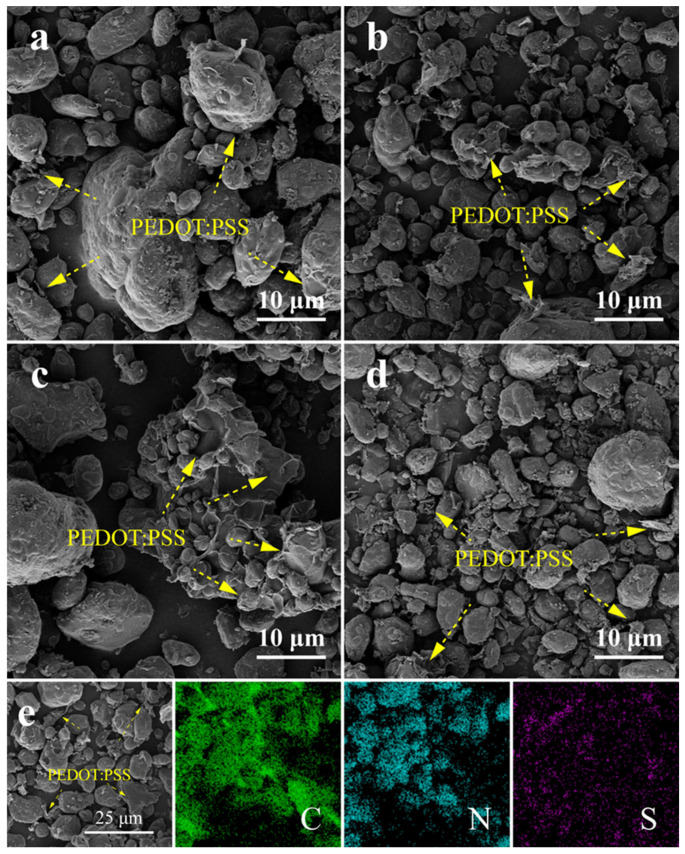
SEM images of (**a**) WP-1, (**b**) WP-3, (**c**) WP-5, (**d**) WP-7, and (**e**) EDS spectra of WP-5.

To further observe the assembly of PEDOT:PSS on the RDX surface, SEM was used for a detailed analysis of the morphology of the composite. The SEM images reveal that the μ-RDX particles exhibit an irregular block or ellipsoidal shape, with a smooth surface covered by small particles (Figure S2a). The morphologies and sizes of these particles did not change after loading with PEDOT:PSS, as confirmed in Figure 1a–d. After loading with 1, 3, 5, and 7 wt% PEDOT:PSS (Figure 1a–d, respectively), the RDX composite particles maintained particle sizes from 5 to 50 μm, with an irregular block shape. Although PEDOT:PSS formed a core–shell structure with most μ-RDX particles, some surfaces of μ-RDX remain exposed. The SEM images indicate that the increase in the PEDOT:PSS content is primarily reflected in the significant increase in the lamellar structures within the composite particles. When 1 wt% PEDOT:PSS was added, it adhered uniformly to the surface of μ-RDX. With the addition of more than 3 wt% PEDOT:PSS, a substantial number of lamellar structures were observed within the composite particles. Considering the presence of the characteristic element S in PEDOT:PSS, EDS spectral analysis confirmed that these lamellar structures were composed of PEDOT:PSS. Notably, although a thin layer of PEDOT:PSS adhered to the surface of μ-RDX particles within the composite, these layers did not form a continuous phase, resulting in the composite particles remaining in a discrete phase. This is because of the pressure differential generated under vacuum conditions during the preparation of the RDX-based composite, which drives the coating of PEDOT:PSS on the RDX surface and the formation of a core–shell structure. However, the large and irregular size of μ-RDX particles leads to damage of the lamellar PEDOT:PSS during the assembly process, resulting in free-standing lamellar PEDOT:PSS. Additionally, the EDS spectra (Figure S2c–e) show that the distribution of C, N, and O on the surface of the composite particles is uniform. The enrichment zones of C, N, and O closely match the contours of the composite particles in the SEM images and likely originate from RDX based on the components of the composite particles. Figure 1e shows the SEM and EDS spectra of WP-5, which show the presence of a distinct PEDOT:PSS coating layer on the surface of μ-RDX particles, along with flake-like PEDOT:PSS between the particles. Comparison of the SEM images with the EDS results reveals that the regions enriched with S closely match the contours of the composites in the SEM images. The S originates from the thiophene rings of PEDOT and the sulfonic acid group of PSS within the PEDOT:PSS structure. The presence of S indicates that PEDOT:PSS is present within the μ-RDX-based energetic composite.

**Figure 2:**
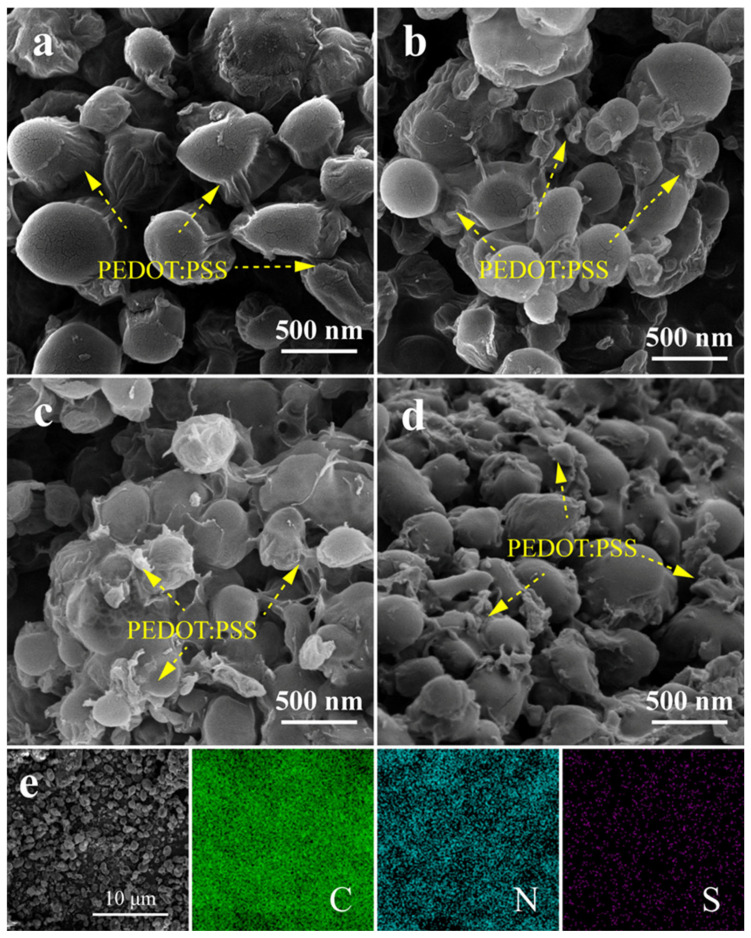
SEM images of (**a**) FP-1, (**b**) FP-3, (**c**) FP-5, (**d**) FP-7, and (**e**) EDS spectra of FP-5.

The raw n-RDX had a small particle size and regular shape, consisting of smooth ellipsoidal particles (Figure S2b). After loading with PEDOT:PSS, the morphology and size of the n-RDX remained unchanged, as confirmed in Figure 2a–d. After loading with 1, 3, 5, and 7 wt% PEDOT:PSS (Figure 2a–d, respectively), the n-RDX composite particles retained sizes of 300–500 nm and their predominantly ellipsoidal shape. The n-RDX-based composite exhibited a jellyfish-like appearance with a noticeable silk-like layer of PEDOT:PSS on the particle surface, indicating that PEDOT:PSS was assembled with RDX under the influence of pressure differences. Unlike the μ-RDX-based composite structure, n-RDX features a typical core–shell structure, where PEDOT:PSS forms a continuous membrane fully wrapping the surface of the n-RDX particles. This is due to the smaller particle size of n-RDX, with a lower volume per particle, allowing the sheet-like PEDOT:PSS to fully encapsulate it, resulting in a core–shell structure with multiple cores (Figure 2c). Moreover, there was no enrichment of S in the n-RDX-based composite; Figure 2e shows the SEM and EDS spectra of FP-5, providing a visual map that clarifies the nature of this material.

The SEM and EDS test results indicate that PEDOT:PSS was successfully coated on the surface of RDX. However, due to unavailability of the required equipment in the laboratory, the elemental analysis of the samples could not be conducted at this stage. This will be focus of the future work.

**Figure 3:**
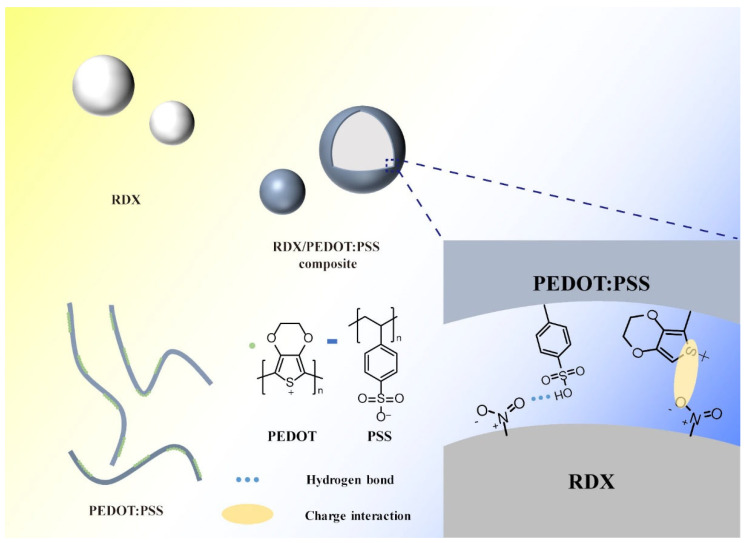
Assembly mechanism of PEDOT:PSS and RDX.

Considering the processes employed in this study and the analysis of the above spectra, we propose the following possible assembly mechanism for PEDOT:PSS (Figure 3). Under the influence of pressure differentials, solid–liquid separation in the suspended RDX/PEDOT:PSS precursor solution occurs rapidly, which drives the solid components together to form the corresponding composite system. Furthermore, since PEDOT:PSS is a polymer composed of positively charged PEDOT groups and negatively charged PSS groups held together by electrostatic interactions [26], the distance between PEDOT:PSS and RDX molecules decreases under pressure differentials, resulting in charge interactions between the S^+^ on the PEDOT thiophene ring and the lone pair of electrons (O^−^) on the oxygen atom of RDX’s nitro group. Concurrently, the sulfonic acid group (-SO_3_H) on the PSS benzene ring forms hydrogen bonds with the oxygen atom (-NO_2_) of RDX’s nitro group. Thus, PEDOT:PSS is coated onto the RDX surface through these synergistic molecular interactions.

### 2.2. Crystal Forms and Molecular Structures Analysis of the RDX-Based Energetic Composites

**Figure 4:**
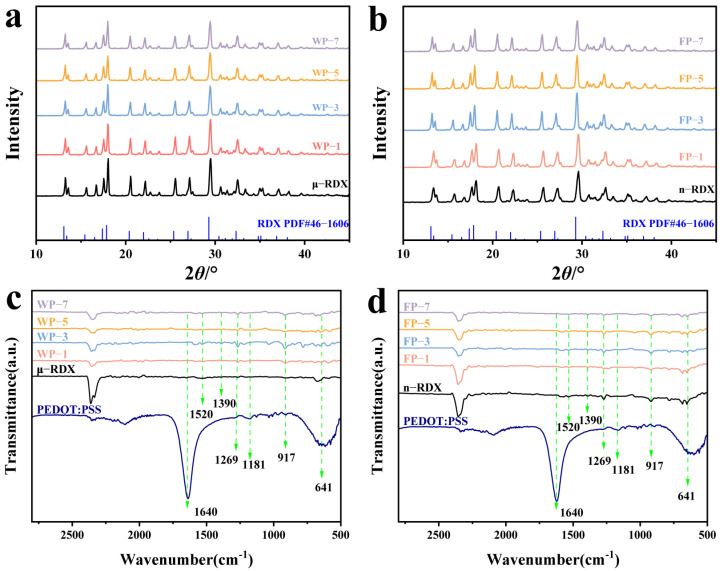
XRD patterns of energetic composites based on (**a**) μ-RDX and (**b**) n-RDX, and FT-IR spectra of energetic composites based on (**c**) μ-RDX and (**d**) n-RDX.

Figure 4a,b presents the XRD patterns of μ-RDX, n-RDX, and their energetic composites. Both μ-RDX and n-RDX exhibit sharp and distinct diffraction peaks, which align closely with the XRD spectra of the RDX composites. In addition, no new characteristic peaks appear in the XRD patterns of the RDX composite particles, indicating that PEDOT:PSS does not affect the crystal forms of RDX during the coating process. However, it is noteworthy that while the positions of the characteristic peaks remain constant, their intensities vary. For instance, at the main characteristic peaks of 13.25°, 17.55°, 18.05°, 20.55°, 22.20°, 25.55°, 27.15°, and 29.5°, which correspond to the crystal planes (1 1 1), (0 2 1), (1 2 1), (2 2 0), (2 1 2), (1 3 1), (1 1 3), and (1 3 2), respectively, the reduction in RDX particle size from micrometers to nanometers results in reduced diffraction peak intensities [27]. Additionally, the diffraction peak intensity of the RDX-based energetic composites decreased significantly, and this phenomenon is more pronounced with an increasing mass fraction of PEDOT:PSS. This diminished peak intensity is primarily due to the coating action of PEDOT:PSS on the crystal surface (Figure 1 and Figure 2) and its amorphous polymer characteristics [4], which weaken the intrinsic diffraction intensity of RDX during XRD testing due to the amorphous anisotropy of PEDOT:PSS and its irregular spatial distribution.

Figure 4c,d shows the infrared spectra of RDX, PEDOT:PSS, and composite particles, where the peak at 1520 cm^−1^ corresponds to the asymmetric stretching vibration of the -NO_2_ group and the peak at 1390 cm^−1^ corresponds to the symmetric stretching vibration of the -NO_2_ group in the RDX crystals [8,28]. The peaks at 1520 and 1269 cm^−1^ are associated with the C–O–C vibration absorption in PEDOT, the peaks at 917 and 641 cm^−1^ correspond to the stretching vibration of the C–S bond in the PEDOT thiophene ring, the peak at 1640 cm^−1^ corresponds to the C=C stretching vibration absorption in PSS, and the peak at 1181 cm^−1^ corresponds to the symmetric stretching vibration of the sulfonic acid group in PSS [29]. The infrared spectra of the composite particles are largely consistent with those of RDX, indicating that the PEDOT:PSS coating process does not affect the inherent molecular structure of the crystals. However, because of the low PEDOT:PSS content of the composites, no characteristic peaks of PEDOT:PSS were detected in the FT-IR spectra. Nonetheless, a slight decrease in the infrared transmittance was observed, indicating that PEDOT:PSS is coated on the RDX surface. The intensity of some absorption peaks in the composite gradually decreased compared to that of RDX, which is attributed to the increasing mass fraction of PEDOT:PSS leading to a gradual weakening of the infrared transmittance of the composite.

### 2.3. DSC Analysis

**Figure 5:**
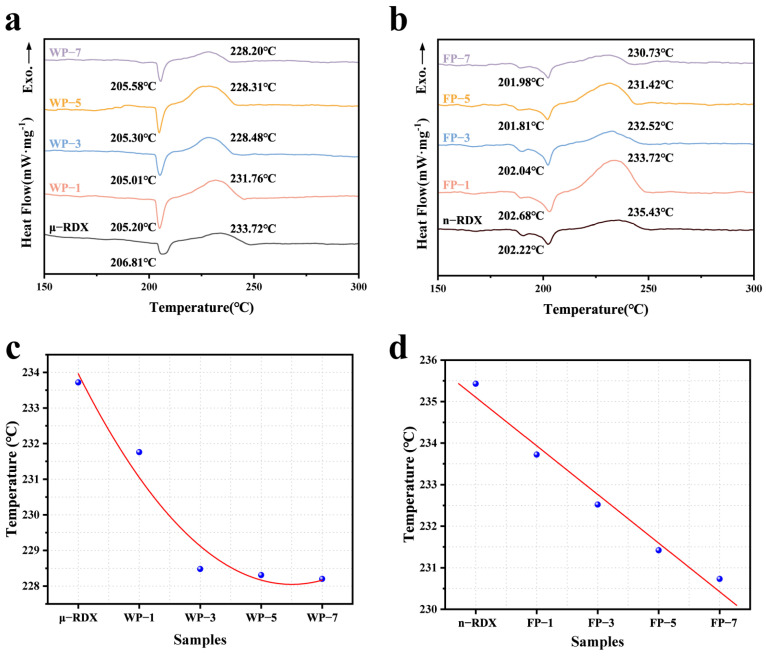
DSC curves of energetic composites based on (**a**) μ-RDX and (**b**) n-RDX, and trend curve of decreasing exothermic peak temperature of energetic composites based on (**c**) μ-RDX and (**d**) n-RDX.

The thermal decomposition properties of RDX and composites were tested using DSC at a heating rate of 10 K·min^−1^ to investigate the effect of PEDOT:PSS on the thermal decomposition of RDX, as shown in Figure 5. It is evident from Figure 5a that μ-RDX has a distinct endothermic peak at 206.81 °C, which corresponds to the phase transition of RDX. Subsequently, as the temperature continues to rise, a strong exothermic peak appears in the DSC curve at 233.72 °C, indicating the thermal decomposition of μ-RDX [30]. Similarly, the RDX-based energetic composite shows only one endothermic and one exothermic peak on the DSC curve, with the endothermic peak occurring between 205 and 206 °C, while the exothermic peak temperature is lower than that of μ-RDX. This indicates that PEDOT:PSS has minimal impact on the endothermic melting process of μ-RDX, with only a slight shift in the position of the endothermic peak. In terms of exothermic behavior, as the mass fraction of PEDOT:PSS increases, the peak temperatures of the composite particles shift forward by 1.96 °C (1 wt%), 5.24 °C (3 wt%), 5.41 °C (5 wt%), and 5.52 °C (7 wt%), indicating that the introduction of PEDOT:PSS promotes the decomposition of μ-RDX. This may be attributed to the following: (1) the occurrence of thermal shock when the nano-PEDOT:PSS coating is heated on the surface of μ-RDX, which increases the friction between the coating and the μ-RDX surface, promoting the formation of local hotspots and accelerating the decomposition of μ-RDX [17]; (2) the molecules of nitramine explosives contain a large number of C–C and N–N bonds, and the initial process of μ-RDX thermal decomposition involves the competitive cleavage of the same type of bonds [31]. The S^+^ in PEDOT easily interacts with the electron-rich nitro group (-NO_2_) in μ-RDX [32], enhancing the electron absorption capability of -NO_2_ and weakening the N–NO_2_ chemical bonds in μ-RDX, making them more susceptible to cleavage during decomposition, thus lowering the activation energy of μ-RDX and facilitating its thermal decomposition. Another noteworthy phenomenon is that the exothermic peak temperature of the composite exhibits an exponential decrease with increasing mass fraction of PEDOT:PSS. The peak temperatures for WP-3, WP-5, and WP-7 are all around 228 °C. This may be due to a stronger interaction between the positive charges of PEDOT:PSS and the lone pair of electrons on the oxygen atom of the nitro group in μ-RDX when the mass fraction of PEDOT:PSS exceeds 1 wt%, resulting in a lower activation energy for μ-RDX.

The endothermic and decomposition peak temperatures of n-RDX in the DSC curve are 202.22 and 235.43 °C, respectively, indicating that the thermal decomposition peak temperature of RDX is higher than that of μ-RDX after ball milling. This is because, as the particle size decreases, the specific surface area and number of surface atoms of the explosive increase, leading to the expansion of the outer electron orbitals and greater vibrational space for atoms, which improves the thermal conductivity between the explosive particles. Consequently, the heat released during decomposition can easily dissipate from the interior of the explosive, making local heat accumulation less likely [33], thus endowing n-RDX with higher thermal stability. Meanwhile, due to the increased specific surface area of n-RDX, the amount of heat absorbed per unit of time increases at a given heating rate, leading to heat concentration. Some n-RDXs begin to undergo a phase transition at around 190 °C, and as the temperature further increases, a significant endothermic peak appears near 190 °C. The DSC curve of the n-RDX-based energetic composite shows a similar decreasing trend in the thermal decomposition peak temperatures. As the mass fraction of PEDOT:PSS increases, the peak temperatures decrease by 1.71 °C (1 wt%), 2.91 °C (3 wt%), 4.01 °C (5 wt%), and 4.70 °C (7 wt%), indicating that PEDOT:PSS promotes the decomposition of n-RDX. However, the exothermic peak temperature decreases linearly with an increasing mass fraction of PEDOT:PSS, and the rate of decrease is slower compared to that of the μ-RDX-based energetic composite. This is due to the higher thermal conductivity of n-RDX, which endows it with a greater ability to conduct heat, resulting in a slight delay in thermal decomposition.

### 2.4. VST Analysis

**Figure 6:**
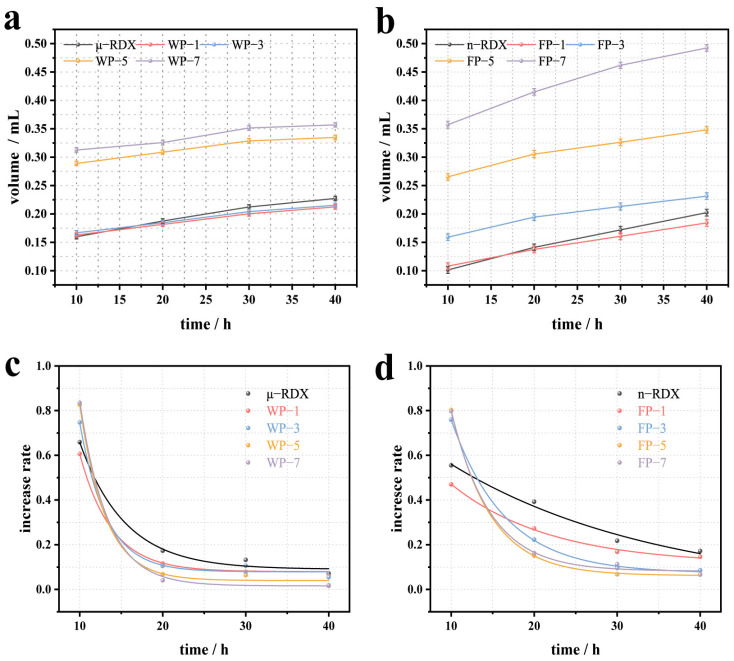
Vacuum stability test (VST) for the gas release volume of energetic composites based on (**a**) μ-RDX and (**b**) n-RDX, and gas release growth rate curves of energetic composites based on (**c**) μ-RDX and (**d**) n-RDX.

To further investigate the thermal decomposition properties of the samples, their thermal stabilities were examined using a VST instrument. Figure 6a shows the VST results for μ-RDX and the corresponding energetic composites. It can be seen that the decomposition and gas release of the μ-RDX sample increase with the testing time, reaching 0.2273 mL after 40 h. For other samples, such as WP-1 and WP-3, the gas release decreased slightly to 0.2124 and 0.2153 mL, respectively, after 40 h of testing. This is due to the influence of so-called “localized chemistry” on the thermal decomposition of RDX, as the PEDOT:PSS film exhibits good thermal stability [34]. When the surface of the RDX particles is coated with PEDOT:PSS, the resulting coating can eliminate or partially eliminate the active decomposition reaction centers on the surface of the RDX crystals, thereby affecting the localized thermal decomposition reaction and promoting the stabilization of the solid RDX crystals. Interestingly, compared to the decrease in the gas release for WP-1 and WP-3, the gas release for the WP-5 and WP-7 samples increased significantly, reaching 0.3347 and 0.3569 mL, respectively, after 40 h. This is primarily because an increase in the PEDOT:PSS content introduces air between it and the RDX [35], which is released upon heating, resulting in increased gas release. Figure 6c presents the fitted curves of gas release growth trends for μ-RDX and the corresponding energetic composites (testing results and fitting equations are given in the Supporting Information, Tables S1 and S3). The gas release amounts at 10 h for μ-RDX, WP-1, WP-3, WP-5, and WP-7 were 0.1598, 0.1628, 0.1669, 0.2888, and 0.3127 mL, respectively, which account for 70.30, 76.65, 77.52, 86.27, and 87.62% of the total gas release, with growth rates of 65.87, 60.64, 74.72, 82.70, and 83.48%, respectively. It can be seen that the main gas release stage for the samples occurs between 0 and 10 h, during which the gas release is highest and the growth rate is fastest. After 20 h, the growth rate decreases sharply and shows a negative correlation with the PEDOT:PSS mass fraction. However, the growth rates of μ-RDX, WP-1, and WP-3 are greater than those of WP-5 and WP-7, possibly because the air in the composites is nearly completely released within 10 h, and the higher content of PEDOT:PSS results in fewer active decomposition reaction centers compared to μ-RDX, WP-1, and WP-3, leading to a smaller growth rate.

Figure 6b shows the VST results for n-RDX and the corresponding energetic composites. The gas release of n-RDX after 40 h is 0.2024 mL, which is lower than that of μ-RDX, indicating a reduction in its decomposition amount. This result is consistent with the DSC test results, suggesting that n-RDX has a higher thermal stability than μ-RDX. Compared with n-RDX, the gas release of FP-1 after 40 h decreased to 0.1841 mL, mainly because of the introduction of PEDOT:PSS, which reduced the probability of forming active decomposition reaction centers in n-RDX and diminished its response to thermal stimuli. Interestingly, the gas released from FP-3, FP-5, and FP-7 after 40 h was higher than that from n-RDX (0.2313, 0.3482, and 0.4924 mL, respectively). It is evident that gas release increases gradually with increasing mass fraction of PEDOT:PSS. This is because the small particle size of n-RDX facilitates the formation of multicore structures during the coating process (Figure 2), resulting in more air within the composite and greater gas release upon heating. Figure 6d presents the fitted curve of the gas release growth rate trends for n-RDX and the corresponding energetic composites (the test results and fitting equations are given in the Supporting Information, Tables S1 and S3). The period from 0 to 10 h was the stage with the highest gas release and the fastest growth rate. However, the thermal conductivity and thermal stability of n-RDX were higher than those of μ-RDX, leading to a slightly lower growth rate during this stage. After 20 h, the growth rate exhibited a similar exponential decay pattern to that of μ-RDX and the corresponding composites.

As shown in Figure 6a,c, the thermal decomposition process of μ-RDX and the corresponding energetic composites during the VST can generally be divided into two stages. The first stage occurs between 0 and 10 h, during which the gas release is the highest and the growth rate of the gas release is the fastest. A detailed analysis revealed that over 88% of the gas was released within the first 5 h of the first stage (Table S2), indicating that this was the main decomposition stage. Therefore, for each sample, the pressure–time curve between 1 and 5 h (Figure S3) was linearized to obtain the slope *k*, which can be used as a measure of the reaction rate (results listed in Table S4). The reaction rate of WP-1 is lower than that of μ-RDX, while the reaction rates of WP-3, WP-5, and WP-7 are higher than that of μ-RDX, which corresponds to the growth rate curve shown in Figure 6c. In the first stage, the PEDOT:PSS content plays a dominant role in the decomposition process. The reaction rate of WP-1 (1 wt%) is lower than that of μ-RDX due to the reduction in active decomposition reaction centers. As the PEDOT:PSS content increases, the reaction rates of WP-3 (3 wt%), WP-5 (5 wt%), and WP-7 (7 wt%) gradually increase, enhancing the thermal reaction activity. A similar trend is observed for n-RDX and the corresponding energetic composites. In the first stage (0–10 h), the reaction rate of FP-1 (1 wt%) is lower than that of n-RDX because of the reduction in active decomposition sites. As the PEDOT:PSS content increases, the slope *k* of FP-3 (3 wt%), FP-5 (5 wt%), and FP-7 (7 wt%) gradually increases, indicating an enhancement in the thermal reaction activity.

### 2.5. Mechanical Sensitivity Analysis

**Figure 7:**
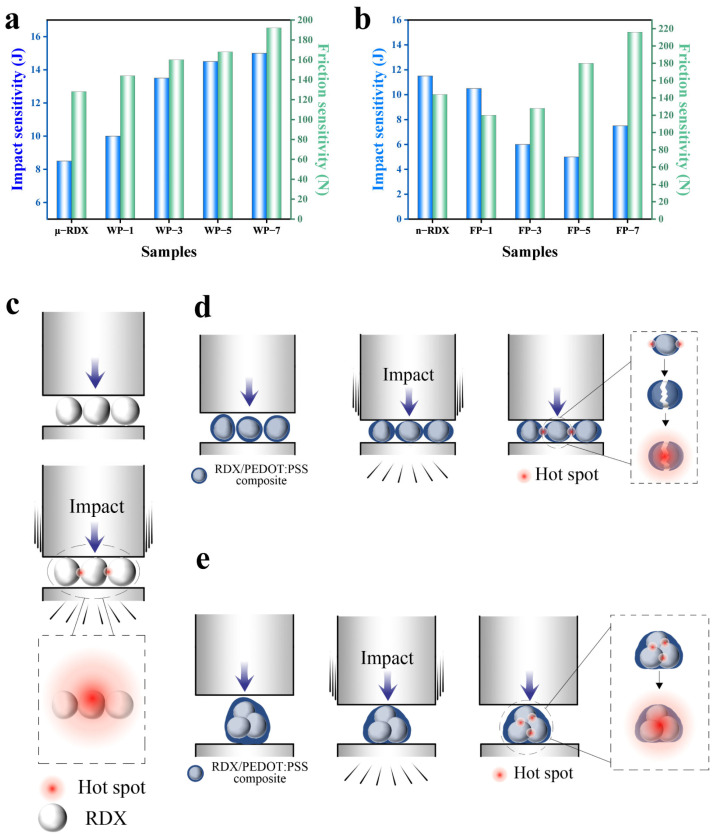
Mechanical sensitivity of energetic composites based on (**a**) μ-RDX and (**b**) n-RDX, and impact explosion schematic of energetic composites based on (**c**) RDX, (**d**) μ-RDX, and (**e**) n-RDX.

The results of the mechanical sensitivity tests examining the impact and friction sensitivities are shown in Figure 7. Impact sensitivity is primarily expressed in terms of the critical impact energy, whereas friction sensitivity is typically evaluated using the limit friction load.

As shown in Figure 7a, the critical impact energy for the explosion of μ-RDX is 8.5 J. Compared to the high impact sensitivity of raw RDX, the critical impact energies of WP-1, WP-3, WP-5, and WP-7 samples, modified with PEDOT:PSS, are 10.0, 13.5, 14.5, and 15.0 J, respectively, showing increases of 1.5 J (17.65%), 5.0 J (58.82%), 6.0 J (70.60%), and 6.5 J (76.47%) over μ-RDX. A similar trend is observed in the friction sensitivity test results. The critical load for ignition of μ-RDX is 128 N, whereas those for WP-1, WP-3, WP-5, and WP-7 are 144, 160, 168, and 192 N, respectively, representing increases of 16 N (12.50%), 32 N (25.0%), 40 N (31.25%), and 84 N (50.0%). From the data above, it can be seen that the critical impact energy and limit friction load of RDX-based energetic composites are positively correlated with the PEDOT:PSS mass fraction. The higher the PEDOT:PSS content, the higher the critical impact energy and limit friction load, indicating that increasing the PEDOT:PSS content can effectively reduce the mechanical sensitivity of RDX. This is mainly due to the fact that when explosive particles are subjected to impact and friction, mechanical actions such as friction, shear, and viscous flow cause the surface temperature to rise, forming localized hotspots. When the hotspot temperature is sufficiently high, it can trigger the RDX redox reaction (Figure 7c) [36,37]. However, after RDX forms a composite with PEDOT:PSS, the presence of PEDOT:PSS between the RDX particles can effectively mitigate the impact force through elastic deformation and absorb the heat generated by the impact, thereby raising the threshold for hotspot formation. This reduces the likelihood of hotspots forming (Figure 7d), thus enhancing the impact and friction safety of the RDX-based energetic composites.

As shown in Figure 7b, the critical impact energy for n-RDX explosion is 11.5 J, and the limit friction load is 144 N. Compared to μ-RDX, its mechanical sensitivity is significantly reduced, which is attributed to the reduction in surface defects after refinement and spheroidization of RDX. A smoother surface with no sharp edges decreases the probability of hotspot formation under impact and friction. On the other hand, the increased specific surface area and surface energy of the n-RDX particles promote particle agglomeration. These agglomerates consume a certain amount of energy when subjected to external mechanical forces, raising the threshold for hotspot formation and further reducing the mechanical sensitivity of n-RDX [38]. Compared to n-RDX, the critical impact energies of FP-1, FP-3, FP-5, and FP-7 decreased by 1.0 J (−8.70%), 5.5 J (−47.83%), 6.5 J (−56.52%), and 4.0 J (−34.78%), respectively, reaching 10.5, 6.0, 5.0, and 7.5 J. This indicates that the impact sensitivity of the composites increased. This is because although the agglomeration of n-RDX was significantly improved after coating with PEDOT:PSS, as the PEDOT:PSS mass fraction increased from 1 to 7 wt%, a large number of voids formed between the particles, and the average void size increased. Under impact, these voids are easily adiabatically compressed, becoming hotspots and increasing impact sensitivity. Notably, FP-7 (7 wt%) exhibited a lower impact sensitivity than FP-1 (1 wt%), FP-3 (3 wt%), and FP-5 (5 wt%) because the higher PEDOT:PSS content provided better cushioning and shielding effects in the coating layer than the other composite particles. Compared to n-RDX, the critical friction loads for ignition of FP-1, FP-3, FP-5, and FP-7 changed by −24 N (−16.7%), −16 N (−11.1%), 36 N (25%), and 72 N (50%), respectively, reaching 120, 128, 180, and 216 N. It can be observed that the friction sensitivity initially increases and then decreases due to ball-milled n-RDX having smaller particle diameters, resulting in a greater contact area and more compact than RDX particles [39]. In addition, the multicore structure formed during the PEDOT:PSS coating results in multiple RDX crystals within the core–shell structure. When the PEDOT:PSS mass fraction is low, the friction between the RDX crystals generates heat, leading to hotspots and thermal accumulation that can cause an explosion. However, when the PEDOT:PSS mass fraction is high, the cushioning and lubricating effects become significant, thereby reducing friction sensitivity.

### 2.6. Electrostatic Spark Sensitivity Analysis

**Figure 8:**
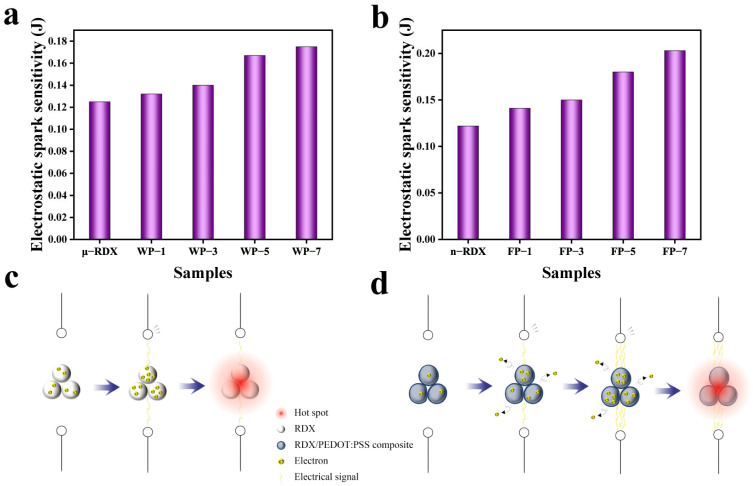
Electrostatic spark sensitivity of energetic composites based on (**a**) μ-RDX and (**b**) n-RDX, and electrostatic stimulus ignition schematic diagram of (**c**) RDX, (**d**) RDX-based energetic composites.

It is well known that RDX is non-conductive. Consequently, when subjected to electrostatic discharge stimulation the surface charge accumulated on RDX can be converted into heat via the Joule effect under the action of an electrostatic spark. This heat increases the local temperature on the surface of the explosive particles, forming hotspots [16]. When the temperature of these hotspots reaches the critical temperature for an explosion, the RDX detonates. Generally, the minimum electrostatic spark energy required to ignite explosive particles is represented by the minimum ignition energy (MIE). Figure 8a,b shows the electrostatic spark sensitivities of the samples used in this study. The ignition energy for μ-RDX is 0.125 J, while for n-RDX it is 0.122 J. It can be observed that the electrostatic spark sensitivity of RDX increases as particle size decreases. This can be attributed to the fact that, in larger explosive particles, the electrostatic spark must follow a longer circular path across the surface of the particles during discharge. As a result, the energy density of the electrostatic spark decreases [40], reducing the electrostatic spark sensitivity of the larger RDX particles.

From Figure 8a, we can see that the MIE values of WP-1 (1 wt%), WP-3 (3 wt%), WP-5 (5 wt%), and WP-7 (7 wt%) increase by 0.007 J (5.6%), 0.015 J (10.7%), 0.042 J (33.6%), and 0.05 J (40.0%), respectively, compared to μ-RDX, reaching 0.132, 0.14, 0.167, and 0.175 J, respectively. As shown in Figure 8b, the electrostatic spark energies of FP-1 (1 wt%), FP-3 (3 wt%), FP-5 (5 wt%), and FP-7 (7 wt%) increase by 0.019 (15.6%), 0.028 (23.0%), 0.058 (47.5%), and 0.081 J (66.4%), respectively, compared to that of n-RDX, reaching 0.141, 0.15, 0.18, and 0.203 J, respectively. After loading RDX with PEDOT:PSS, the MIE values of the composite particles are higher, and the electrostatic spark sensitivity is reduced. Figure 8c,d reveals the possible desensitization mechanism of PEDOT:PSS on RDX. PEDOT:PSS is a conductive polymer and after assembly with RDX, a conductive interface is formed on its surface. This conductive interface provides the composite system with an efficient path for electrostatic charge conduction, preventing charge accumulation on the surface of the RDX. When the composite particles are stimulated by electrostatic sparks, the PEDOT:PSS coating layer isolates and protects the RDX crystals from the direct impact of the spark energy, thereby preventing surface heating and explosion [8]. For the composite particles, the core–shell-like structure of PEDOT:PSS significantly reduces the electrostatic spark sensitivity of RDX. Moreover, the PEDOT:PSS content directly affects the desensitization effect. The electrostatic spark sensitivity of the composite particles decreases as the PEDOT:PSS mass fraction increases, with the electrostatic spark energy increasing from 5.6 to 66.4%. For the same mass fraction of PEDOT:PSS coating on μ-RDX and n-RDX, the desensitization effect is more significant for n-RDX. This is because the surface of μ-RDX remains partially exposed after coating with PEDOT:PSS and a continuous conductive network is not formed, making the exposed areas more susceptible to direct electrostatic spark impact and charge accumulation. In contrast, the smaller particle size of n-RDX allows PEDOT:PSS to completely encapsulate the RDX particles, as observed in the SEM results. The surface of the n-RDX forms a continuous PEDOT:PSS coating layer, providing better encapsulation and allowing for more effective electrostatic charge conduction and protection.

## 3. Experimental

### 3.1. Materials

Micron-sized RDX with an average particle size of approximately 40 μm was provided by Gansu Yin Guang Chemical Industry Group Co., Ltd., Baiyin, China. Nanosized RDX with an average particle size of approximately 500 nm was prepared using a mechanical ball-milling method [41]. Conductive grade PEDOT:PSS solution (1.3 wt% aqueous dispersion) was obtained from Sigma-Aldrich, Shanghai, China. Deionized water was purchased from Lanquan Water Industry Co., Ltd., Xuchang, China.

### 3.2. Preparation of RDX-Based Energetic Composites

**Figure 9:**
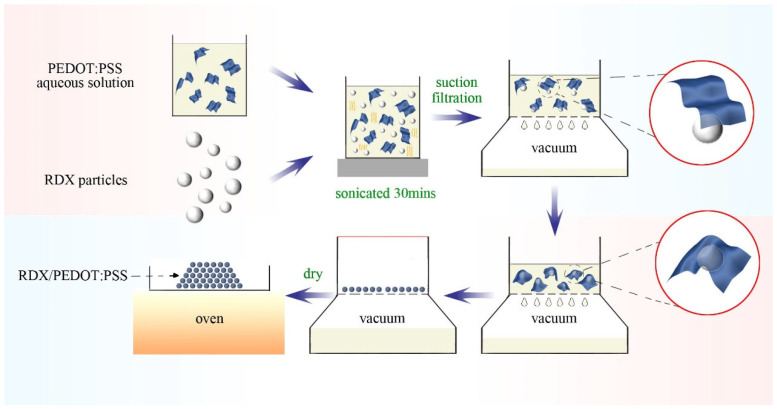
Schematic illustration of preparation of RDX-based energetic composites.

The process used to prepare the RDX-based energetic composites is illustrated in Figure 9. A certain amount of RDX was added to a beaker containing deionized water, and then ultrasonically dispersed using a 35 watt ultrasonic disperser for 10 min to form a uniform white suspension. Subsequently, 1 wt%, 3 wt%, 5 wt%, and 7 wt% aqueous solutions of PEDOT:PSS were pipetted into the beaker, and the mixture was further ultrasonically dispersed for 30 min to ensure uniform dispersion of the suspension. The suspension was then rapidly passed through a nylon filter membrane (pore size 0.45 μm) under differential pressure, and the RDX-based composites were obtained after vacuum drying at 55 °C. During this process, both micron-sized RDX (μ-RDX) and nano-sized RDX (n-RDX) were used, resulting in eight types of RDX-based energetic composites prepared with PEDOT:PSS proportions of 1, 3, 5, and 7 wt%; the composites based on μ-RDX are labeled as WP-1, WP-3, WP-5, and WP-7, respectively, whereas the n-RDX-based energetic composites are labeled FP-1, FP-3, FP-5, and FP-7, respectively.

### 3.3. Structural and Morphological Characterization of RDX-Based Energetic Composites

The microscopic morphology and surface elemental distribution of the RDX-based energetic composites were analyzed using a scanning electron microscope (SEM; MIRA3 TESCAN, Brno, Czech Republic) and an energy-dispersive X-ray spectrometer (EDS; X-max 80, Oxford Instruments, Concord, MA, USA). The crystal structure of the RDX-based energetic composites was examined using an X-ray diffractometer (XRD; DX-2700, Dandong Haoyuan Instruments Co., Ltd., Dandong, China) with a tube voltage of 40 kV, a tube current of 30 mA, and a testing angle of 5° to 50° with a 0.05° step angle. The molecular structure of the energetic composites was analyzed using a Fourier-transform infrared spectrometer (FT-IR; Invenio S, Bruker Optics, Karlsruhe, Germany), with a testing range of 4000 to 500 cm^−1^ and a resolution of 4 cm^−1^.

### 3.4. Response Characteristics Tests of RDX-Based Energetic Composites

The decomposition performance of RDX-based energetic composites under thermal stimulation was tested using a differential scanning calorimeter (DSC; DSC-800B, Yingnuo Instruments Co., Ltd., Shanghai, China), with a sample mass of 0.7 mg, a temperature range of 50–350 °C, a heating rate of 10 K·min^−1^, and a flowing nitrogen atmosphere at a flow rate of 30 mL·min^−1^. The thermal stability of the RDX-based energetic composites was tested using a vacuum stability tester (VST, Stabil VI, OZM Research, Prague, Czech Republic), with a sample mass of 50 mg at a testing temperature of 100 ± 0.5 °C for 40 h.

The friction sensitivity of the samples was tested using a friction sensitivity tester (BAM FSKM-10, OZM Research, Prague, Czech Republic) under the following conditions: ambient temperature of 20–25 °C, relative humidity ≤ 40%, and a sample volume of approximately 10 mm^3^. The impact sensitivity of the samples was evaluated using an impact sensitivity tester (BAM BFH-12, OZM Research, Prague, Czech Republic) under the following conditions: ambient temperature of 20–25 °C, relative humidity ≤ 40%, a sample volume of approximately 40 mm^3^, and a drop weight of 2.5 kg.

The electrostatic spark sensitivities of the samples were tested using an electrostatic spark sensitivity tester (BAM Xspark10, OZM Research, Prague, Czech Republic) [42]. The voltage and capacitance during ignition were measured using a step-up method to calculate the minimum electrostatic spark energy required for 100% explosion probability.

## 4. Conclusions

To reduce the RDX electrostatic spark and mechanical sensitivities and improve safety performances, this study selected conductive polymer PEDOT:PSS as a coating material to prepare RDX-based energetic composites by constructing a conductive interface on the RDX crystals surface. SEM and EDS tests showed that PEDOT:PSS assembled with RDX under the combined effects of pressure difference, electrostatic forces, and hydrogen bonding. XRD and FT-IR analyses indicated that during the assembly process, the crystal forms and molecular structure of RDX remained constant. DSC and VSTs demonstrated that PEDOT:PSS enhanced the thermal reactivity of RDX, with the peak decomposition temperature advancing by up to 5.52 °C as the PEDOT:PSS proportions increased. The mechanical sensitivity of μ-RDX-based energetic composites was significantly improved, with increased impact safety by 1.5–6.5 J (17.65–76.47%) and decreased friction sensitivity by 16–84 N (12.5–50.0%). The impact sensitivity of n-RDX-based energetic composites increased, with the maximum value of critical impact energy decreasing by 6.5 J (56.52%), while the friction sensitivity showed an initial increase followed by a decrease, with a maximum increase of critical load by 72 N (50%). The electrostatic spark sensitivity of μ-RDX-based energetic composites decreased by 0.007–0.05 J (5.6–40%), while that of n-RDX-based energetic composites decreased by 0.019–0.081 J (15.6–66.4%). Moreover, the electrostatic spark sensitivity decreased with increasing PEDOT:PSS content, indicating that the introduction of conductive interfaces successfully endowed the composite particles with excellent electrostatic safety performance. Therefore, this preparation strategy for RDX/PEDOT:PSS composites undoubtedly provides a promising approach for the safe application of RDX.

## Data Availability

The data presented in this study are contained within the Supplementary Materials.

## Supplementary Materials

The following supporting information can be downloaded at: https://www.mdpi.com/article/10.3390/molecules30051000/s1, Figure S1: Picture of samples; Figure S2: SEM of (a) μ-RDX; (b) n-RDX and EDS images of (c) WP-1; (d) W-3; (e) WP-7; (f) FP-1; (g) FP-3; (h) FP-7; Figure S3: Recordings of gas pressure during 0–10 h at 100 °C of energetic composites based on (a) μ-RDX and (b) n-RDX; Table S1: Gas release volumes of samples; Table S2: Gas release volumes of samples in 0–10 h; Table S3: Samples increase rate fitting equations; Table S4: Samples VST pressure-time curve linearization parameters.
