# Supplementary material for: Construction and Performance Characterization of Hexahydro-1,3,5-trinitro-1,3,5-triazine/Poly(3,4-ethylene-dioxythiophene)–Poly(styrenesulfonate) Energetic Composites
Source: Molecules. 2025 Feb 21;30(5):1000. doi: 10.3390/molecules30051000 (PMC11901766; doi:10.3390/molecules30051000)
Supplement: Supplementary file 1 [file molecules-30-01000-s001.zip › molecules-3395560-supplementary.pdf]

Construction and Performance Characterization of Hexahydro-1,3,5-trinitro-1,3,5-triazine/Poly(3,4-ethylene-dioxythiophene) – Poly(styrenesulfonate) Energetic Composites

Zhiwei He [1,*], Gongzhen Zhang [2], Chuanhao Xu [3,*], Wenyu Zhu [4], Jiawei Yue [1], Shengtao Zhou [1] and Zhenyi Huang [1]

1School of Chemical and Blasting Engineering, Anhui University of Science and Technology, Huainan 232001, China; 2022201175@aust.edu.cn(J.Y.); 2022201166@aust.edu.cn(S.Z.); 2022201224@aust.edu.cn(Z.H.)

2Henan Huatong Chemical Industry Co., Ltd., Xinyang 465200, China; 13865542329@163.com(G.Z.)

3School of Environment and Safety Engineering, North University of China, Taiyuan 030051, China

4Anhui Jiangnan Chemical Industry Co., Ltd., Hefei 230094, China; 17775210572@163.com(W.Z.)

*Correspondence: zhwhe@aust.edu.cn(Z.H.); xuchuanhao@yeah.net(C.X.)

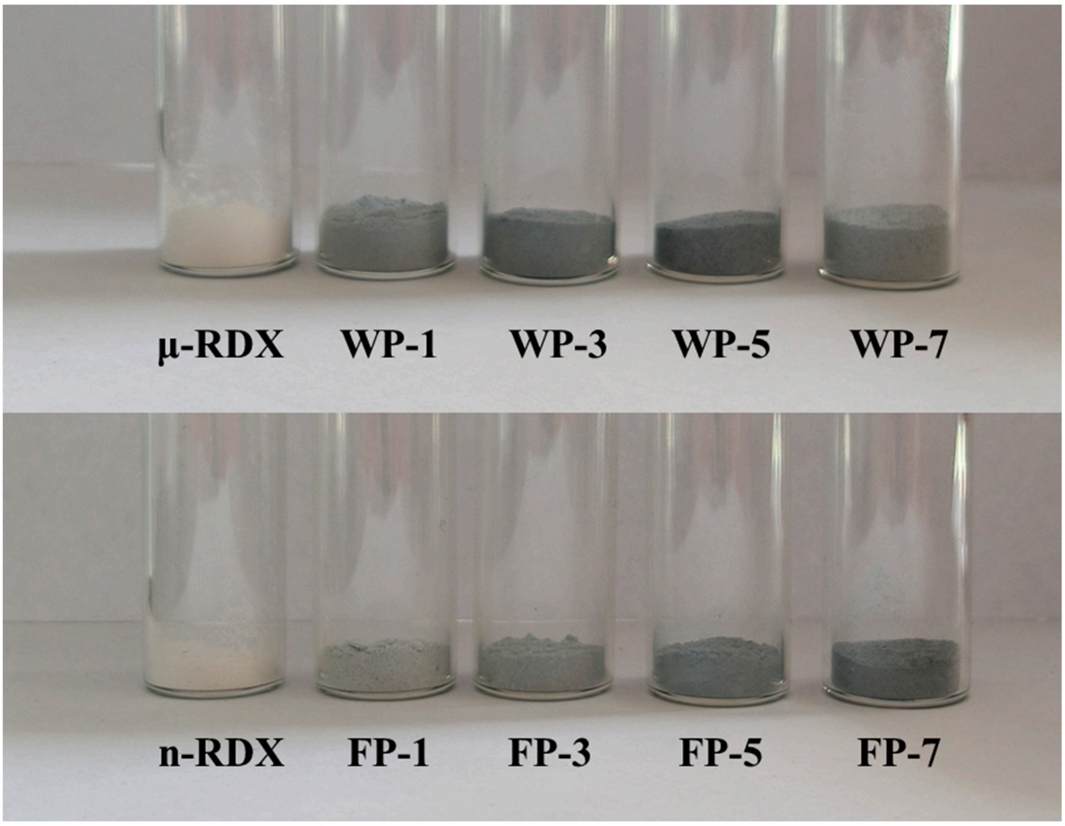

Figure S1 Picture of samples

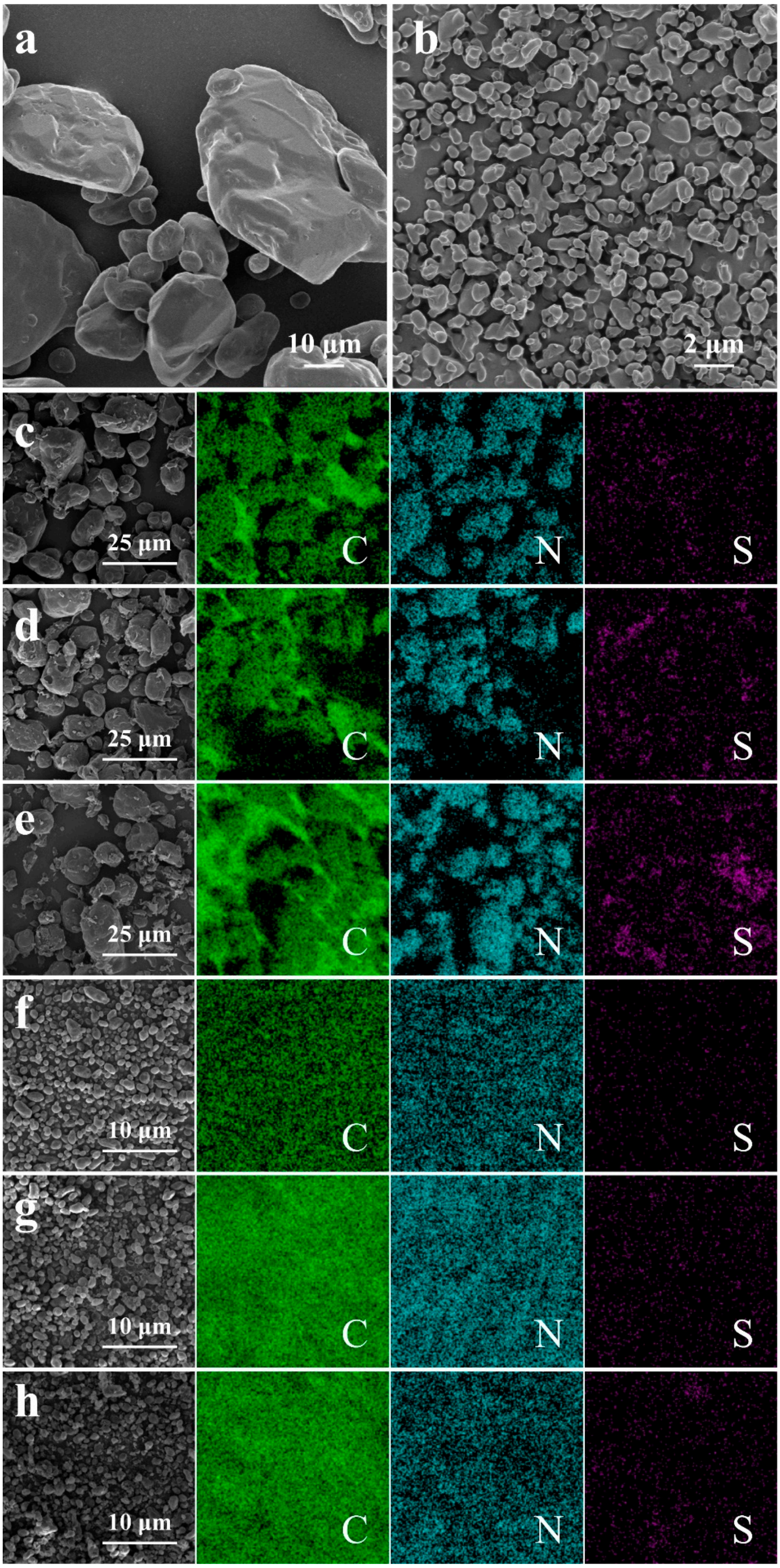

Figure S2 SEM of (a) µ-RDX; (b) n-RDX and EDS images of (c) WP-1; (d) W-3; (e) WP-7; (f) FP-1;(g) FP-3; (h) FP-7

Table S1 Gas release volumes of samples

time / h	0	10	20	30	40
	volume / mL	volume / mL	volume / mL	volume / mL	volume / mL
μ-RDX	0.0545	0.1598	0.1876	0.2123	0.2273
WP-1	0.0641	0.1628	0.1816	0.2003	0.2124
WP-3	0.0422	0.1669	0.1845	0.2041	0.2153
WP-5	0.0499	0.2888	0.3088	0.3286	0.3347
WP-7	0.0517	0.3127	0.3257	0.3514	0.3569
n-RDX	0.0451	0.1014	0.1411	0.1718	0.2024
FP-1	0.0573	0.1080	0.1376	0.1607	0.1841
FP-3	0.0383	0.1591	0.1945	0.2132	0.2313
FP-5	0.0523	0.2653	0.3055	0.3264	0.3482
FP-7	0.0721	0.3572	0.4148	0.4617	0.4924

Table S2 Gas release volumes of samples in 0-10 h

time / h	3	4	5	6	7
	volume / mL	volume / mL	volume / mL	volume / mL	volume / mL
μ-RDX	0.1194	0.1270	0.1351	0.1418	0.1462
WP-1	0.1358	0.1419	0.1479	0.1520	0.1552
WP-3	0.1412	0.1498	0.1539	0.1568	0.1600
WP-5	0.2611	0.2670	0.2731	0.2791	0.2803
WP-7	0.2679	0.2731	0.2761	0.2798	0.2829
n-RDX	0.0645	0.0706	0.0768	0.0825	0.0884
FP-1	0.0818	0.0863	0.0906	0.0945	0.0975
FP-3	0.1242	0.1330	0.1389	0.1452	0.1487
FP-5	0.2192	0.2312	0.2409	0.2467	0.2529
FP-7	0.3030	0.3148	0.3235	0.3324	0.3384

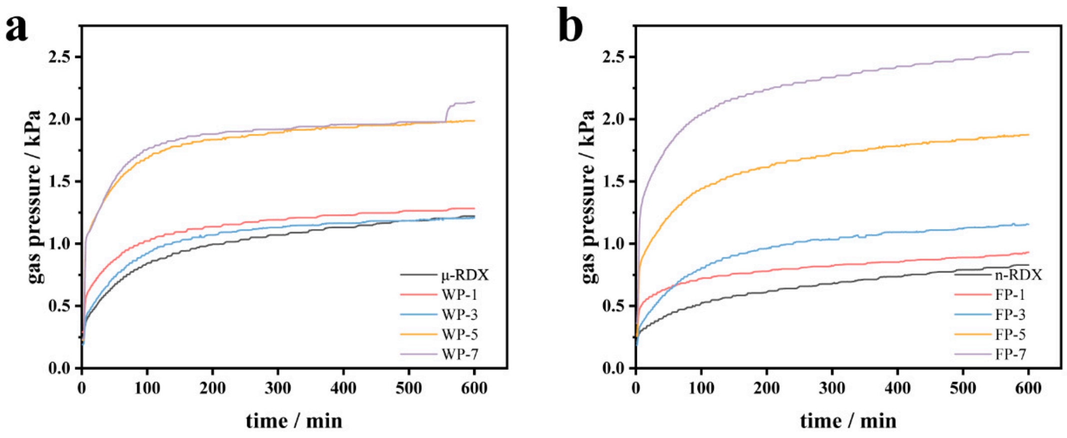

Figure S3 Recordings of gas pressure during 0-10 h at 100°C of energetic composites based on (a) μ-RDX and (b) n-RDX

Table S3 Samples increase rate fitting equations

Samples	Fitting Equation	R^2
μ-RDX	$y=3.56263e^{-\frac{x}{5.44675}}+0.09007$	0.98257
WP-1	$y=7.21431e^{-\frac{x}{3.82072}}+0.07965$	0.98527
WP-3	$y=15.3167e^{-\frac{x}{3.19284}}+0.07889$	0.98837
WP-5	$y=20.5259e^{-\frac{x}{3.06632}}+0.03999$	0.99355
WP-7	$y=2617.85049e^{-\frac{x}{1.23357}}+0.04535$	0.98711
n-RDX	$y=0.85389e^{-\frac{x}{25.03793}}-0.01251$	0.95663
FP-1	$y=0.83159e^{-\frac{x}{11.90997}}+0.11182$	0.9924
FP-3	$y=3.21029e^{-\frac{x}{6.48469}}+0.07278$	0.99888
FP-5	$y=6.22711e^{-\frac{x}{4.6977}}+0.06208$	0.99971
FP-7	$y=6.09577e^{-\frac{x}{4.66915}}+0.082$	0.9938

Table S4 Samples VST test pressure-time curve linearization parameters

Samples	Equation of linearization		
	Slope/*k*	Intercept	R^2
μ-RDX	0.00353±4.73088E-5	1.95327±0.00854	0.95796
WP-1	0.00305±5.17398E-5	2.47285±0.00951	0.93457
WP-3	0.00362±7.03915E-5	2.15208±0.01250	0.91503
WP-5	0.00752±1.69689E-4	4.65180±0.03080	0.88837
WP-7	0.00699±1.88316E-4	5.04902±0.03443	0.84801
n-RDX	0.00162±1.72108E-5	1.51806±0.00318	0.97336
FP-1	0.00129±1.64448E-5	1.91691±0.00308	0.96238
FP-3	0.00356±5.21237E-5	1.87470±0.00938	0.95008
FP-5	0.00794±1.06786E-4	3.39759±0.01899	0.95756
FP-7	0.01576±2.28419E-4	6.06693±0.04034	0.95089
